# Animal experimental investigation on the efficacy of antibiotic therapy with linezolid, vancomycin, cotrimoxazole, and rifampin in treatment of periprosthetic knee joint infections by MRSA

**DOI:** 10.1302/2046-3758.113.BJR-2021-0268.R1

**Published:** 2022-03-01

**Authors:** Julia Goetz, Verena Keyssner, Frank Hanses, Felix Greimel, Franziska Leiß, Timo Schwarz, Hans-Robert Springorum, Joachim Grifka, Jens Schaumburger

**Affiliations:** 1 Department of Orthopaedic Surgery, University Hospital Regensburg - Asklepios Bad Abbach, Regensburg, Germany; 2 Department of Infectology, University Hospital Regensburg, Regensburg, Germany

**Keywords:** MRSA, Infection of the artificial joint, Combination antibiotics therapies, Antibiotics monotherapy, Vancomycin, Rifampin or cotrimoxazole, Linezolid and rifampin, antibiotic therapies, vancomycin, rifampicin, methicillin-resistant Staphylococcus aureus (MRSA), joint infections, strains, antibiotics, rat model, femur, clinical studies

## Abstract

**Aims:**

Periprosthetic joint infections (PJIs) are rare, but represent a great burden for the patient. In addition, the incidence of methicillin-resistant *Staphylococcus aureus* (MRSA) is increasing. The aim of this rat experiment was therefore to compare the antibiotics commonly used in the treatment of PJIs caused by MRSA.

**Methods:**

For this purpose, sterilized steel implants were implanted into the femur of 77 rats. The metal devices were inoculated with suspensions of two different MRSA strains. The animals were divided into groups and treated with vancomycin, linezolid, cotrimoxazole, or rifampin as monotherapy, or with combination of antibiotics over a period of 14 days. After a two-day antibiotic-free interval, the implant was explanted, and bone, muscle, and periarticular tissue were microbiologically analyzed.

**Results:**

Vancomycin and linezolid were able to significantly (p < 0.05) reduce the MRSA bacterial count at implants. No significant effect was found at the bone. Rifampin was the only monotherapy that significantly reduced the bacterial count on implant and bone. The combination with vancomycin or linezolid showed significant efficacy. Treatment with cotrimoxazole alone did not achieve a significant bacterial count reduction. The combination of linezolid plus rifampin was significantly more effective on implant and bone than the control group in both trials.

**Conclusion:**

Although rifampicin is effective as a monotherapy, it should not be used because of the high rate of resistance development. Our animal experiments showed the great importance of combination antibiotic therapies. In the future, investigations with higher case numbers, varied bacterial concentrations, and changes in individual drug dosages will be necessary to be able to draw an exact comparison, possibly within a clinical trial.

Cite this article: *Bone Joint Res* 2022;11(3):143–151.

## Article focus

- Our research group established a rat model to simulate infection of the artificial joint. A sterile implant was drilled into the femur and two different bacterial suspensions were used, methicillin-resistant *Staphylococcus aureus* (MRSA) strains ATCC 43300 and COL.
- The controlled animal study compares the efficiency of antibiotic monotherapies with vancomycin, linezolid, cotrimoxazole or rifampin and the combination with rifampin in periprosthetic joint infections (PJIs) with the MRSA pathogen in the knee joint.

## Key messages

- Rifampin was the only monotherapy that led to a significant bacterial count reduction in bone and implant, but with a high rate of resistance development (p < 0.05).
- The combination of vancomycin or linezolid with rifampin has significantly better efficacy (reduction of MRSA bacterial count) on bone and implant than the respective monotherapy. For this reason, combination therapy plays a major role in the treatment of periprosthetic MRSA infection.

## Strengths and limitations

- A strength of the study is the controlled animal design.
- Another strength is the fact that this is the first study which compares different monotherapy and combination antibiotic therapies.
- The limitations are the small case number and the monocentric study design. In addition, this is only an animal study in which one joint was examined.

## Introduction

Every year, approximately 150,000 hip and knee arthroplasties are carried out in the UK.^1^ Reasons for failure of joint arthroplasty therapy are septic loosening due to periprosthetic joint infection (PJI), and aseptic loosening due to abrasion, periprosthetic ossification, or arthrofibrosis.^2^

Because of major advances in surgical techniques, implant material, and hygiene awareness, the incidence of PJI has been significantly reduced and is currently between 1% and 2%.^3^ However, if repeated revision surgery is necessary, the risk increases to 20%.^4^ Factors that increase infection are alcohol, nicotine abuse, heart failure, tumour disease, radiation, immune deficiency or immunosuppressive drugs, diabetes, obesity, post-traumatic arthritis, rheumatoid arthritis or periprosthetic fractures.^5^

A distinction is made between exogenous and haematogenous infections. Different classifications of PJIs are found in the literature. However, they are classified according to the time of infection and the type of infection path. Exogenous bacterial colonization can occur perioperatively through contamination of the implant, gloves, the patient’s skin, or through germs that migrate into the surgical site from neighbouring foci, such as skin and soft-tissue lesions or osteomyelitis. Haematogenous infections can take place at any time. Common primary foci are skin lesions, urinary tract infections, respiratory infections, diverticulitis or preceding colonoscopy, dental abscesses, and bile duct inflammation.^6^ In 57% of cases, no primary focus can be found.^7^ Trampuz and Zimmerli^8^ published the following classification: an early postoperative infection is present if it occurs less than three months after joint arthroplasty. It is often caused by highly virulent bacteria (such as *Staphylococcus aureus* or gram-negative bacteria), with a frequency of 29% to 45%. A delayed or low-grade infection has a frequency of 23% to 41%. This is often caused by less virulent germs such as coagulase-negative staphylococci. The late (chronic) infection manifests more than two years after joint arthroplasty with a frequency of 30% to 33%. The cause of the late infection is haematogenic. The entry ports are usually skin injuries, respiratory tract, teeth, or urinary tract.

Shortly after methicillin was introduced as a drug against staphylococci, methicillin-resistant *Staphylococcus aureus* (MRSA) was first isolated and described in a European hospital in 1961.^9^ Responsible for methicillin resistance is the mec-gene, which contains mecA, mecI, and mecRI. MRSA mainly colonizes the nasal area and skin, and also, less frequently, the urinary tract and the anal area. The rate of therapy failures in the treatment of MRSA infections has now increased nine-fold and is 38%. As a result, patients with MRSA infections stay longer in hospital (on average 15 days) and have higher morbidity and mortality, which leads to significantly higher costs. The systemic antibiotics are decisive for the success of the therapy.^10,11^

Zimmerli et al^12^ showed in 1984 that an inoculum of only 10^2^ colony-forming units (CFUs) *Staphylococcus aureus* was sufficient to infect an implant, whereas 10^5^ CFUs bacteria were required without foreign material. Overall, in animal experiments it can be assumed that at least 10^3^ CFUs, and on average 10^5^ to 10^8^ CFUs, are required for 100% infection development.

The aim of this study is to compare the efficiency of antibiotic therapy with vancomycin, linezolid with, and linezolid without rifampin in PJIs with the MRSA pathogen in an in vivo model.

## Methods

The controlled animal study was performed after approval by the local and state animal protection committee. All animal experiments were carried out in accordance with the EU Directive 2010/63/EU. All ARRIVE guidelines were adhered to and the checklist was supplied. The study used 82 male Wistar rats, of which 77 were included in the analysis. At the beginning of the study, the animals were aged between 12 and 14 weeks and had a mean body weight of 397.3 g (standard deviation (SD) 19.83). After veterinary examination by the head of the central animal laboratory and animal welfare officer, the animals were kept in individual cages. The temperature of 21°C (±1°C), a mean humidity level of approximately 55% (45% to 75%), and an air exchange rate of 15 air exchanges per hour was predefined. An automated artificial lighting system provided the 12-hour day-night rhythm with 12 hours of brightness (300 lux) and 12 hours of darkness. After delivery, the animals had seven days of acclimatization before the experiment started. All interventions and drug applications were performed in the same intervention room of the animal house.

**Fig. 1:**
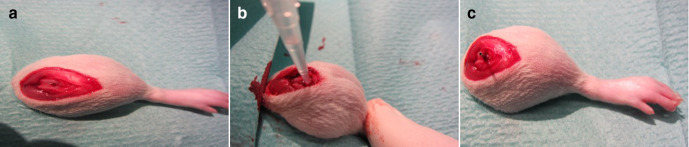
a) Drill hole in distal femur. b) Methicillin-resistant *Staphylococcus aureus* injection into drilled femur. c) Implanted sterile steel implants into rat femur.

Our research group established a rat model, which has already been used for preliminary experiments on tissue and plasma concentrations of linezolid and experiments with methicillin-sensitive *Staphylococcus aureus.* ^13^ The surgical implantation was performed under deep anaesthesia according to the principle of retrograde femoral nailing under sterile conditions. For this purpose, the animals were injected with 6 to 8 mg/kg xylazine 2% and 90 to 120 mg/kg ketamine 10% intraperitoneally, and the left hind leg was carefully shaved. After thorough disinfection and sterile covering, a skin incision of 1 cm was made medially of the patella and the knee joint was prepared. The patella was dislocated laterally, the femoral joint was located, and the intercondylar medullary space was reamed retrogradely by using a hand drill (Figure 1).

In accordance with a previously published study, a sterile steel implant made of steel with a length of 1.5 cm and a diameter of 1.0 mm was used as an implant.^13^ The implant was then implanted retrogradely into the distal femur. Finally, the medullary cavity was sealed with bone wax, the soft-tissue was closed layer by layer with single button sutures, and the skin was stapled. Two different bacterial suspensions were used. First, bacteria of the strain MRSA ATCC 43300 were used. In experiment 2, pathogens of the strain COL were used. Both strains are internationally recognized reference strains of methicillin-resistant staphylococci. In the end, 45 implants were infected with bacteria of the MRSA strain ATCC 43300 and a bacterial concentration of 10^9^ CFUs/ml. The remaining 32 experimental animals were injected with a suspension of the MRSA strain COL, with a bacterial count concentration of 5×10^8^ CFUs/ml. In order to give the PJI time to establish itself, the therapy was started seven days after surgery. The animals were injected with either sterile water, linezolid, vancomycin, rifampin, cotrimoxazole or a combination of rifampin and one of the other antibiotics (Table I). In the COL trial, however, the groups rifampin, cotrimoxazole, and cotrimoxazole with rifampin were not used.

**Table I.** Number of animals divided into the following groups: L = Linezolid, V = Vancomycin, R = Rifampin, C = Cotrimoxazole, W = sterile water, depending on test with methicillin-resistant *Staphylococcus aureus* strains ATCC 43300 or COL.

Test	L	V	R	C	W	LR	VR	CR
ATCC 43300	6	6	6	5	6	6	6	4
COL	6	6	0	0	6	7	7	0

Antibiotic administration was carried out twice per day at 6am and 6pm, and was always intraperitoneal. Rifampin was administered only at 6am, whereas all other drugs were administered twice daily. The antibiotic solutions were prepared according to the body weight of the animals. On day 0, 7, 14, 21, and 23 the animals were weighed, which provided the opportunity to follow and document the effects of surgery and antibiotic therapy.

On the 23rd study day, the animals were anaesthetized by intraperitoneal injection of 90 to 120 ml/kg ketamine 10% and 6 to 8 ml/kg xylazine 2%, and killed by overdosing pentobarbital into the heart. The dead laboratory animals were weighed and placed in disinfectant solution for a few minutes to prevent contamination by remaining germs. The surgical removal of the implants was carried out under sterile conditions. First, the skin of the left hind leg was opened with a scalpel. Then periarticular soft-tissue and muscle tissue were removed. All tissue samples were shock-frozen in cryo-tubes in liquid nitrogen and stored at -80°C until microbiological evaluation. The microbiological evaluation of results was carried out at the Institute for Medical Microbiology and Hygiene. In the first step, tissue samples were ground using a micro-dismembranator for two minutes at 2,500 rotations/min, weighed, and then placed in solution in 4 ml of sterile 0.85% sodium chloride. After, they were stirred at 250 stirs/min for one minute. To remove the biofilm, the implant was first placed in an ultrasonic bath in a watertight sealable tube containing 4 ml of 0.85% sodium chloride for 15 minutes. Subsequently, mechanical cleaning was performed with the addition of glass beads for one minute at 250 vortex-induced vibrations/min. The quantitative determination of CFUs in the tissue and biofilm suspension was analyzed with semi-automatic spiral plater. This automatically aspirates the suspension, so that 50 µl each was plated out on two Mueller-Hinton plates. In addition, 50 µl and 200 µl of suspension were manually applied to a blood agar plate. Before and after each plating out of a sample suspension, 100 µl of sterile 0.85% sodium chloride was plated out twice on each Mueller-Hinton plate to detect any contamination. Between processing different samples, the automatic cleaning cycle of the spiral plater was performed. Subsequently, all agar plates were incubated bottom up at 35°C (±2°C) for two days. After 24 and 48 hours of incubation, the number of CFUs was counted according to the instructions, and with the help of the counting template belonging to the spiral plater. The detection limit for bacteria was 80 CFUs; samples with lower bacterial counts were considered sterile (CFU = 0). To prove that the growing bacteria were indeed *S. aureus*, the pathogen was tested for catalase and coagulase positivity.

### Statistical analysis

Descriptive statistics included number and percentage for categorical variables, and mean, standard deviation for numerical variables. The independent two-group comparisons of the numerical variables were performed using independent-samples *t*-test when the normal distribution condition was provided, and Mann-Whitney U test was performed when the normal distribution condition was not provided. Statistical significance level of α was accepted as p < 0.05. “P” describes the probability with which the null hypothesis “The two groups are equal” is erroneously rejected.

## Results

The first test was infection with the MRSA strain ATCC 43300, to determine and compare the antimicrobial effect of vancomycin, linezolid, cotrimoxazole, rifampin, and their combination with rifampin.

Osteomyelitis was established in all 45 animals. This was controlled by tissue sample during explantation of the implants. Already on the first postoperative day the animals showed usual drinking and eating behaviour. Some animals showed a partial weightbearing posture of the operated leg, but apart from that they moved freely in their cage. Wound healing was unproblematic, although some of the animals removed their skin clips by gnawing themselves.

All animals showed a weight loss between 4.62% and 8.85% on day 7. However, during the antibiotic therapy, the animals gained weight progressively and almost reached their initial weight at the end of the treatment. In the linezolid (L), rifampin (R), and sterile water (W) groups the initial weight was even exceeded. In the antibiotic-free interval, before implant removal, body weight decrease was recorded in the groups cotrimoxazole (C), cotrimoxazole/rifampin (CR), and linezolid/rifampin (LR). The rats of the other study groups had weight gain. Animals in the antibiotic groups suffered more from diarrhoea than the control group. The rifampin-treated rats showed a distinct orange colouration of urine and stool.

In a comparison of the monotherapies, rifampin showed the best bacterial count reduction in implant-associated infections with the MRSA strain ATCC 43300. The analyses of the various tissue samples and the implant determined bacterial counts below the detection limit. Linezolid reduced the bacterial count in bone, muscle, and periarticular tissue more than vancomycin (bone, p = 0.937; muscle, p = 0.662; and periarticular tissue, p = 0.394, Mann-Whitney U test), and even more than cotrimoxazole (bone, p = 0.429; muscle, p = 0.662; and periarticular tissue, p = 0.610, Mann-Whitney U test), but without significance. In contrast, vancomycin (p = 0.905, Mann-Whitney U test) led to stronger reduction of bacterial count at the implant than linezolid.

**Fig. 2:**
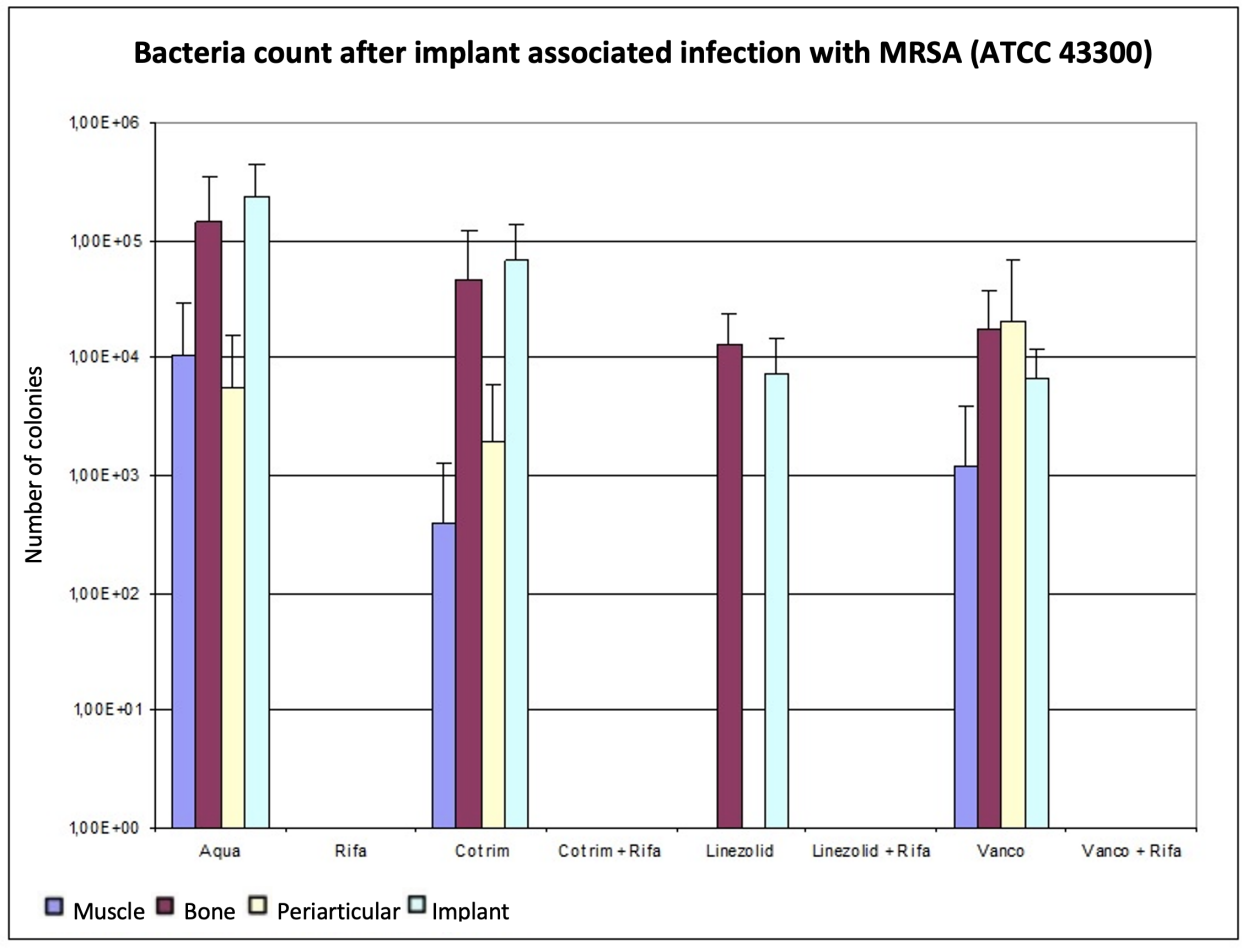
Determination of the bacterial count in tissue with methicillin-resistant *Staphylococcus aureus* (MRSA) (ATCC 43300), expressed as mean value and standard deviation. The bacterial count is expressed in the tissue in colony-forming units (CFUs)/g and in the implant in CFUs/Implantat. The detection limit is 80 CFUs.

In comparison with cotrimoxazol, vancomycin showed a better reduction in the bacterial count on both bone and implant. Conversely, on muscle and periarticular tissue, cotrimoxazole showed fewer CFUs/g than vancomycin. The combination therapies reduced the bacterial count in all tissues below the detection limit. Figure 2 and Table II provide an overview of the results.

**Table II.** ATCC 43300: comparison of bacterial counts after therapy on bone, implant, muscle tissue, and periarticular tissue, expressed in p-values using Mann-Whitney U test.

Variable	C + R	L	L + R	V	V + R	W	R
**Bone**							
C	0.016	0.429	0.004	0.537	0.004	0.690	0.004
C + R		0.010	1.000	0.038	1.000	0.016	1.000
L			0.002	0.937	0.002	0.429	0.002
L + R				0.015	1.000	0.004	1.000
V					0.015	0.329	0.015
V + R						0.004	1.000
W							0.004
**Implant**							
C	0.200	0.200	0.024	0.095	0.024	0.143	0.024
C + R		0.200	1.000	0.071	1.000	0.095	1.000
L			0.024	0.905	0.024	0.036	0.024
L + R				0.002	1.000	0.004	1.000
V					0.002	0.004	0.002
V + R						0.004	1.000
W							0.004
**Muscle tissue**							
C	0.730	0.662	0.662	1.000	0.662	0.177	0.662
C + R		1.000	1.000	0.730	1.000	0.114	1.000
L			1.000	0.662	1.000	0.065	1.000
L + R				0.662	1.000	0.065	1.000
V					0.662	0.247	0.662
V + R						0.065	1.000
W							0.065
**Periarticular tissue**							
C	0.686	0.610	0.610	0.762	0.610	0.730	0.610
C + R		1.000	1.000	0.476	1.000	0.413	1.000
L			1.000	0.394	1.000	0.329	1.000
L + R				0.394	1.000	0.329	1.000
V					0.394	1.000	0.394
V + R						0.329	1.000
W							0.329

C, cotrimoxazole; L, linezolid; R, rifampin; V, vancomycin; W, sterile water.

When monotherapies were compared, only rifampin showed a significant bacterial count reduction of the MRSA strain ATCC 43300 on bone (p < 0.05). There were no significant differences within the other monotherapies. Each of the three combination therapies was significantly more effective than cotrimoxazole or linezolid or vancomycin as a monotherapy (p < 0.050). Linezolid plus rifampin, or vancomycin plus rifampin, were equally effective and tended to be more effective than cotrimoxazole in combination with rifampin. However, this difference was not significant (p > 0.050) (Table II). Rifampin lowered the bacterial count significantly below the detection limit on bone (p < 0.050). Additionally, the combination therapies achieved bacterial counts significantly below the detection limit on bone compared to the control group (p < 0.050). Within the combination groups, no differentiation was possible.

The bacterial counts after therapy on implant, which were also determined using the Mann-Whitney U test, showed that linezolid and vancomycin as well as rifampin delivered significantly lower values than the control group (p < 0.050). In direct comparison with linezolid and vancomycin, no significant difference was found (p = 0.905). The combinations of vancomycin, as well as linezolid, with rifampin significantly reduced the bacterial count compared to monotherapy (p < 0.05). The combination therapy of cotrimoxazole and rifampin showed no significant reduction of MRSA bacterial count at the implant compared to the control group (p = 0.095, Mann-Whitney U test).

No significant differences were found between therapies in muscle tissue after infection with the MRSA strain ATCC 43300 (Table II). Combination therapies and monotherapy with linezolid and rifampin resulted in pathogen numbers below the detection limit; cotrimoxazole tended to be more effective than vancomycin.

In periarticular tissue (Table II), we also detected no significant differences between therapies (p > 0.05). As in muscle, the microbiological analysis showed no bacterial growth in periarticular tissue in combination therapy groups, or in monotherapies with rifampin and linezolid. Cotrimoxazole was similar or more effective in bone (p = 0.537), muscle (p = 1.00), periarticular tissue (p = 0.762), and implant (p = 0.095, all Mann-Whitney U test) compared to vancomycin, but without significance. Figure 2 shows that after conducting and evaluating the experiment with ATCC 43300, the combination therapies VR and LR reduced the MRSA count in all tissues below the detection limit of 80 CFUs.

The second test was infection with the MRSA strain COL, to determine and compare the antimicrobial effect of vancomycin and linezolid, and their combination therapy with rifampin. The experimental procedure was identical to trial 1, except that the groups cotrimoxazole, rifampin, and cotrimoxazole plus rifampin were omitted. The 32 animals were distributed among the groups sterile water, linezolid, vancomycin, linezolid plus rifampin, and vancomycin plus rifampin.

In all groups, a weight loss between 3.6% and 7.7% was recorded on day 7. However, during the antibiotic therapy, the animals gained weight progressively. The animals treated with linezolid plus rifampin had almost regained their initial weight on day 21. In the linezolid, sterile water, and vancomycin study groups, the initial weight was exceeded. In the vancomycin plus rifampin group, a decrease in body weight was observed until the end of the experiment. With the exception of the sterile water control group, the experimental animals lost weight in the antibiotic-free interval before the implant was removed.

**Fig. 3:**
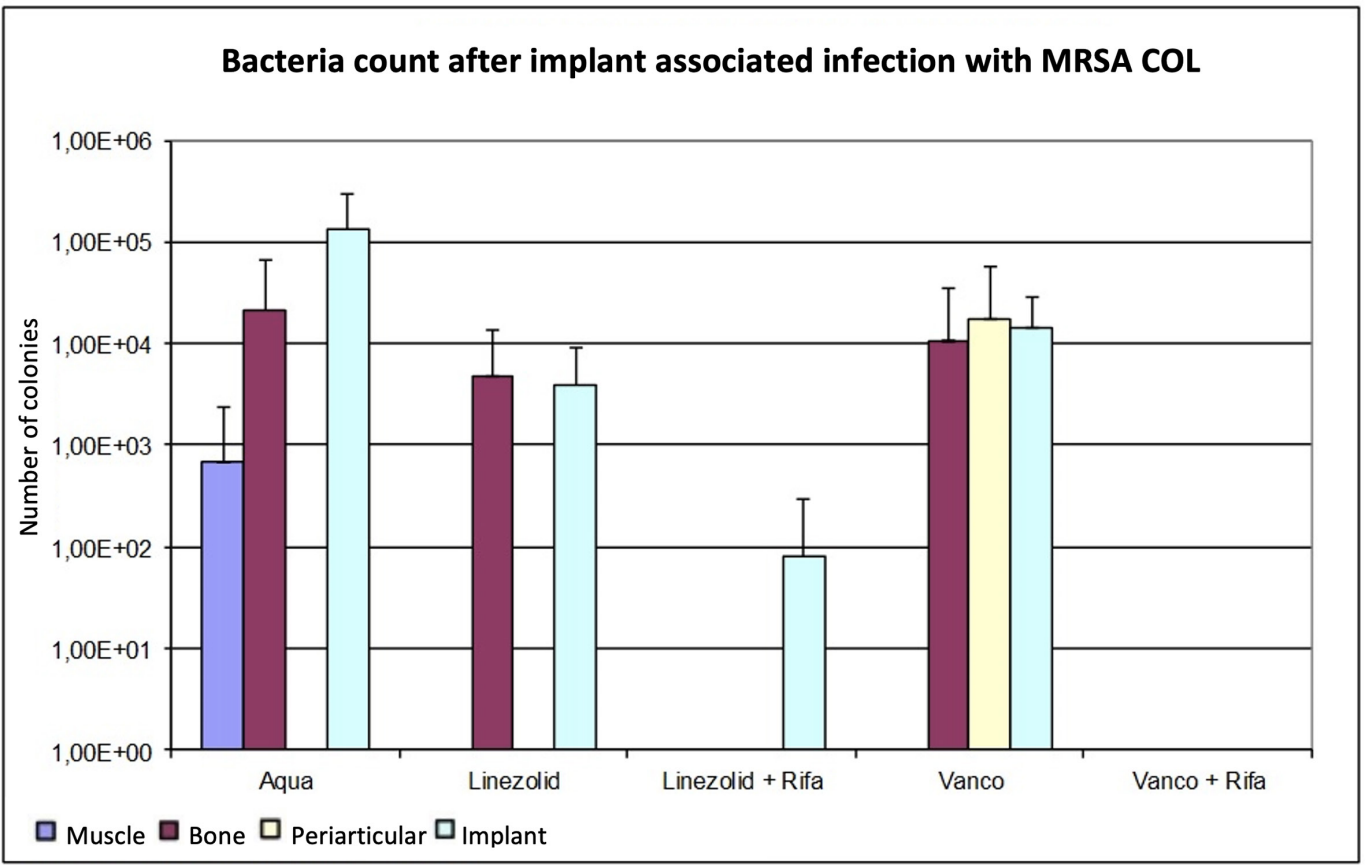
Determination of the bacterial count in tissue with methicillin-resistant *Staphylococcus aureus* (MRSA) (COL), expressed as mean value and standard deviation. The bacterial count is expressed in the tissue as colony-forming units (CFUs)/g and in the implant in CFU/Implantat. The detection limit is 80 CFUs.

As with ATCC 43300, the combinations with rifampin achieved better results than monotherapy. Linezolid provided lower bacterial counts in all tissues compared to control group and vancomycin, but only at implants with significant results (p < 0.05, Mann-Whitney U test). The combined treatment of vancomycin with rifampin resulted in bacterial counts below the detection limit in all tissues. The combination of linezolid plus rifampin also reduced the bacterial counts below the detection limit, except for the implant, where a low mean bacterial count was detected. Despite the change of the MRSA strain, no further differentiation of the microbiological efficacy between the combination therapies could be discerned. An overview of the results is shown in Figure 3 and Table III.

**Table III.** Comparison of bacterial counts after therapy on bone, implant, muscle tissue, and periarticular tissue using Mann-Whitney U test.

Variable	LR	V	VR	W
**Bone**				
L	0.042	1.000	1.000	0.886
LR		0.230	1.000	0.042
V			0.257	0.686
VR				0.067
**Implant**				
L	0.026	0.24	0.015	0.03
LR		0.015	0.699	0.004
V			0.015	0.177
VR				0.004
**Muscle tissue**				
L	1.000	1.000	1.000	0.662
LR		1.000	1.000	0.628
V			1.000	0.699
VR				0.699
**Periarticular tissue**				
L	1.000	0.699	1.000	1.000
LR		0.628	1.000	1.000
V			0.699	0.699
VR				1.000

C, cotrimoxazole; L, linezolid; R, rifampin; V, vancomycin; W, sterile water.

As in trial 1, the p-value for bone in regard to the monotherapies was not significant (p > 0.05, Mann-Whitney U test). In comparison with the monotherapies, the effect of linezolid was better than vancomycin, although without significance (p > 0.05, Mann-Whitney U test). The combination therapies reduced the bacterial counts below the detection limit, whereby only the combination of linezolid and rifampin was significant (p > 0.05). On the implant, microbiological analysis showed that linezolid had a significant effect compared to the control group (p = 0.030, Mann-Whitney U test). Vancomycin as monotherapy did not cause a significant reduction of pathogen count on the implant (p = 0.177, Mann-Whitney U test). As Table II shows, the combination therapies produced significantly lower pathogen counts compared to control group and monotherapies (p < 0.05). In a direct comparison, vancomycin plus rifampin was slightly more effective than the combination with linezolid, but without significance (p = 0.699, Mann-Whitney U test).

In reference to Table III, there were no significant differences between muscle and periarticular tissue after the different antibiotic therapies (p > 0.05, Mann-Whitney U test). In periarticular tissue, the MRSA bacterial count in the control group was not detectable; only in the vancomycin group was a pathogen count detectable.

## Discussion

Animal experimental models are generally accepted for comparing different therapies in the case of implant-associated osteomyelitis,^14,15^ as they allow for more complex interactions between host, pathogen, and foreign material compared to in vitro experiments. Larger animals such as rabbits^16^ or dogs^17^ are preferred, because their size allows the implantation of knee prostheses and thus better imitation of the situation in humans. However, the surgical procedure for these animals is complex and expensive. Rats, on the other hand, are more cost-effective in terms of acquisition, maintenance, and tissue analysis. Furthermore, complications of long-term antibiotic therapies, such as pseudomembranous colitis,^18^ are less frequent. Sclerosing substances were not used in order to replicate the environment of the human body as closely as possible. The use of sterile steel implants in arthroplasty is also described in the literature, especially for rats.^19^

The treatment and classification of PJIs are major issues in the literature. Revised classifications based on tumour classification, such as the PJI­-TNM system,^20,21^ allow treatment guidelines for specific situations. The prophylaxis of MRSA infections, e.g. by ethylenediaminetetraacetic acid wound irrigation, is also being further researched.^22^ In the event of MRSA infection, antibiotic therapy is the treatment of choice.

Only vancomycin showed a significant reduction in bacterial count on the implant in the ATCC 43300 trial. By contrast, no significant effect could be observed in the trial with COL. The reduction was also above the significance level in bone, muscle, and periarticular tissue. This result supports the findings of a systematic review of the literature from 2020, which included 4,607 patients.^23^ In primary knee arthroplasty, intrawound vancomycin reduces the incidence of PJI, but it may increase the risks of aseptic wound complications. For this reason, we opted for intraperitoneal application.^24^ Saleh-Mghir et al^25^ investigated the efficacy of vancomycin in a rabbit model. Partial prostheses with tibial components were implanted into the right knee of the animals, inoculated with MRSA, and then treated intramuscularly with 60 mg/kg vancomycin or 30 mg/kg quinupristin-dalfopristin, with and without rifampin, over a period of seven days. They could demonstrate a significant reduction of MRSA bacterial count by using quinupristin-dalfopristin. In combination with rifampin, the results were significantly better than the control or monotherapy groups. As is the case with our trial, this group showed no significant treatment success of vancomycin on the implant. However, the shorter therapy duration of one week must be taken into account.

Several clinical studies report the successful administration of linezolid in the case of PJI. Rao et al^26^ demonstrated success in the linezolid treatment of osteomyelitis with and without implant; the pathogens were MRSA*,* methicillin-resistant coagulase-negative staphylococci, vancomycin-resistant *Enterococcus faecium*, and vancomycin-sensitive *Enterococcus faecalis*. According to Li et al,^27^ linezolid is significantly better than even vancomycin in the case of MRSA infection. An open-label, randomized, comparator-controlled, multicentre, multinational study confirmed that significantly more patients, 87% after linezolid and in 48% after vancomycin, were microbiologically cured. Clinically, there was also an improvement in the linezolid group, however this effect was not statistically significant. Our experimental tests can confirm these observations on the implant: linezolid showed a significant bacteria count reduction on the implant in both ATCC 43300 and COL MRSA strains.

Rifampin was the only monotherapy to significantly reduce the bacterial count in implant-associated infections with the MRSA strain ATCC 43300 in bone, and at the implant, below detection limit. In comparison, Vergidis et al^28^ published a study in which titanium wires inoculated with MRSA were implanted into the tibia of rats. As in our experiments, rifampin caused a significant reduction in the bacterial count in bone and titanium implant compared with the control group. However, some development of resistance was detected in 63% of the rifampin animals group, which could be considerably reduced in combination with vancomycin or linezolid. Additionally, John et al^29^ showed that rifampin had a significant effect on foreign body-associated infections with MRSA, but with frequent development of resistance – this could be reduced by the administration of vancomycin. Their data confirm our results, namely that rifampin has significant efficacy against MRSA in implant-associated osteomyelitis, and should not be administered as a monotherapy due to high-resistance development.

In studies such as the one by Neyisci et al,^30^ a vancomycin-loaded VK100 silicone cement drug delivery system seems to be an effective method for the treatment of implant-related chronic MRSA osteomyelitis in rats. Additionally, Kaur et al’s^31^ examination of the efficacy of linezolid presents an attractive and aggressive early approach in preventing, as well as treating, implant-associated infections caused by MRSA strains. Linezolid is also a cost-effective drug and offers the advantage of oral administration.

In our study, the combined administration of linezolid plus rifampin significantly reduced the number of pathogens on the implant and bone in both experiments. Vancomycin plus rifampin also showed a significant difference on bone in trial one with ATCC 43300, but not with *MRSA* strain COL. The additional administration of vancomycin or linezolid made it possible to significantly reduce the development of resistance compared to rifampin monotherapy. The rabbit model used by Saleh-Mghir et al^32^ achieved similar results: the combined administration of vancomycin with rifampin proved to be significantly more effective than vancomycin monotherapy.

Along the same lines as our study, Baldoni et al^33^ showed that the combination of linezolid and rifampin is significantly more effective than linezolid monotherapy. There was no significant difference between the linezolid administration doses of 25 mg/kg, 50 mg/kg, and 75 mg/kg. Garrigós et al^34^ achieved similar results in their study.

On bone, the combined administration of cotrimoxazole and rifampin showed a significantly better effect compared to the control group and the cotrimoxazole monotherapy. Conversely, there was no significant difference when compared to rifampin. Nguyen et al^35^ researched the effects of combined administration of cotrimoxazole and rifampin on bone and joint inflammation with *S. aureus*. With a success rate of 78.6% for the administration of cotrimoxazole and rifampin, there was no significant difference compared to linezolid plus rifampin (89.3%).

Overall, our study affirms the importance of combination antibiotic therapies. The reasons for our investigation are to broaden the spectrum of activity and prevent resistance mechanisms from evolving; enhancing intracellular penetration and using synergistic effects play a major role in this.^36^ A limitation of the study, in addition to the relatively small number of cases, is that risk of infection recurrence was not evaluated. Yang et al^37^ describe a recurrence risk of 18.5% after PJI. In the future, antimicrobial coatings could play a role in preventing PJI.^38-40^

In conclusion, the study has proven to be suitable for the evaluation of different antibiotic regimes in periprosthetic MRSA infections of knee joints. Rifampin was the only monotherapy that led to a significant bacterial count reduction in bone and implant. For this reason, it is a key component in the treatment of periprosthetic MRSA infection. However, rifampin should not be administered as monotherapy due to a high rate of resistance development. We have shown that the combination of vancomycin or linezolid with rifampin is significantly more effective on bone and implant than the respective monotherapy. A differentiation comparison of the efficacy of both combination antibiotic treatments was not possible despite the change of MRSA strain, because the bacterial counts were below the detection limit even in the second test. In addition, the effect of linezolid was better (without significance) than vancomycin and cotrimoxazole, with the advantage that linezolid can be taken orally. Nevertheless, further studies with larger case numbers are necessary to investigate the efficacy of mono and combination antibiotic treatments, and to see whether the dosage of rifampin can be decreased in combination therapy.
